# A Case of Lymphocytic Myocarditis with Eosinophilic Degranulation Successfully Treated with Steroid Therapy

**DOI:** 10.1155/2020/8887726

**Published:** 2020-07-28

**Authors:** Tomoko Inoue, Fusako Sera, Shunsuke Nishimura, Kei Nakamoto, Yasumasa Tsukamoto, Isamu Mizote, Tomohito Ohtani, Shungo Hikoso, Yoshihiko Ikeda, Yumiko Hori, Hatsue Ishibashi-Ueda, Eiichi Morii, Tetsuo Minamino, Yasushi Sakata

**Affiliations:** ^1^Department of Cardiovascular Medicine, Osaka University Graduate School of Medicine, Osaka, Japan; ^2^Department of Cardiorenal and Cerebrovascular Medicine, Faculty of Medicine, Kagawa University, Kagawa, Japan; ^3^Department of Pathology, National Cardiovascular Center, Osaka, Japan; ^4^Department of Pathology, Osaka University Graduate School of Medicine, Osaka, Japan

## Abstract

A 49-year-old woman was admitted with suspicion of acute myocarditis. On the next day after admission, her serum troponin I level continued to rise, indicating progression of myocardial damage. Moreover, her symptoms persisted, and left ventricular ejection fraction did not improve. Because of a predominant infiltration of lymphocytes in the myocardial specimens, lymphocytic myocarditis was diagnosed. However, a close observation of the specimens revealed eosinophil degranulation. Based on this finding, intravenous steroid therapy was initiated. High-dose methylprednisolone led to rapid and appreciable improvements in symptoms and left ventricular function within 12 hours after the first administration, which was followed by normalization of serum troponin I level. Steroid therapy was switched to oral administration and tapered carefully. There was no recurrence of left ventricular dysfunction or elevation of serum troponin I level. In eosinophilic myocarditis, eosinophil degranulation has been recognized as an important finding associated with progression of inflammation and myocardial damage. However, no attention has been paid to the presence and clinical implications of eosinophil degranulation in lymphocytic myocarditis. This case indicates that eosinophil degranulation in lymphocytic myocarditis may be an important finding associated with a high therapeutic response to steroid therapy.

## 1. Introduction

Acute myocarditis has a broad spectrum of clinical presentations, ranging from mild symptoms to severe heart failure and lethal arrhythmia requiring mechanical circulatory support [1]. In some cases, delayed diagnosis and intervention may result in fatal hemodynamic deterioration. The therapeutic strategy for acute myocarditis varies according to histological changes; therefore, early endomyocardial biopsy should be considered to guide cause-specific treatment [2]. Immunosuppressive therapy such as steroids plays an important role in eosinophilic and giant cell myocarditis; however, its efficacy remains controversial in lymphocytic myocarditis [3].

We herein report a case of acute lymphocytic myocarditis successfully treated with steroid therapy, in which a predominantly lymphocytic infiltration of the myocardium was accompanied by eosinophil degranulation.

## 2. Case Presentation

A 49-year-old woman presented to our hospital with arthralgia, nausea, and fever lasting for seven days and chest pain over a few days. Laboratory data showed elevated serum troponin I level. Echocardiography showed reduced left ventricular (LV) ejection fraction (LVEF) with anterior wall motion abnormalities. The patient was admitted to our hospital because myocardial infarction or myocarditis was suspected.

She had no significant medical history or known drug allergies. On admission, chest pain, nausea, and inappetence persisted. Although blood pressure values were normal, she had physical fatigue and sinus tachycardia with minimal exertion. Laboratory data on admission showed a white blood cell count of 4460/*μ*L, including 7.2% eosinophils (eosinophil count: 321/*μ*L), serum creatine kinase (CK) level of 147 U/L (CK-MB: 3.7 ng/mL), troponin I level of 0.47 ng/mL, and B-type natriuretic peptide (BNP) level of 109 pg/mL. The levels of immunoglobulin and autoantibody were within normal limits.

**Figure 1:**
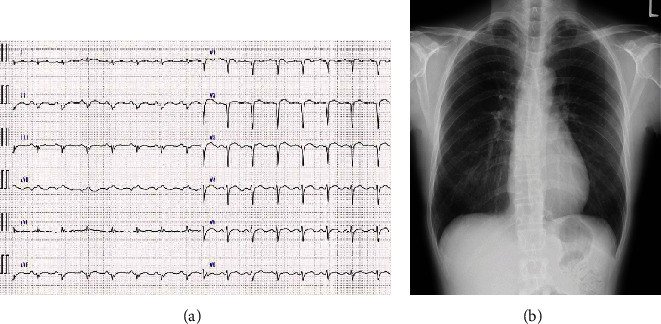
Electrocardiography and thoracic radiography findings on admission. (a) Electrocardiogram shows a sinus rhythm with poor R progression in precordial leads; (b) postero-anterior thoracic radiogram shows no features of heart failure.

The electrocardiogram showed sinus rhythm with low voltage in limb leads and poor R progression in precordial leads (Figure 1(a)). Thoracic radiography excluded cardiomegaly, pleural effusion, or pulmonary congestion (Figure 1(b)). Echocardiography revealed a LV end-diastolic diameter of 50 mm and LVEF of 42% with hypokinesis in the LV anterior region.

**Figure 2:**
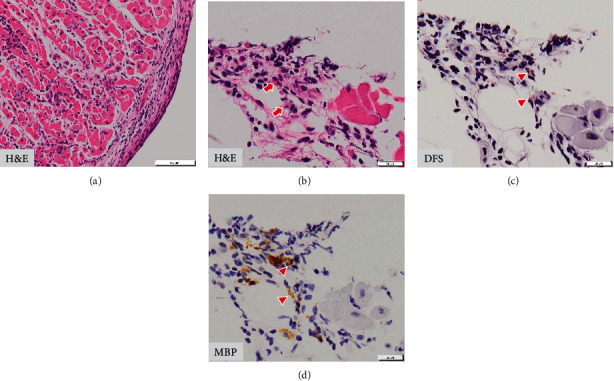
Right ventricular myocardial biopsy specimen. (a) Extensive inflammatory cell infiltration (consisting of lymphocytes and no giant cells) with little necrosis (hematoxylin-eosin (H&E) staining); (b) a few eosinophils (red arrow) with degranulation can be observed (H&E staining); (c, d) eosinophil degranulation (red arrowhead) confirmed by direct fast scarlet (DFS) staining and immunostaining for major basic protein (MBP).

Coronary angiography showed no significant stenosis. Three endomyocardial biopsy specimens were obtained from the right interventricular septum. Myocardial specimens showed abundant lymphocytic infiltration with interstitial edema and slight perivascular fibrosis (Figure 2(a)). Very few eosinophils and no giant cells were found in the specimens. Based on these findings, she was pathologically diagnosed with acute lymphocytic myocarditis. However, a close observation of the specimens revealed eosinophils with degranulation (Figure 2(b)). Eosinophil degranulation was also confirmed by direct fast scarlet stain (DFS) and immunostaining for major basic protein (MBP) (Figures 2(c) and 2(d)).

**Figure 3:**
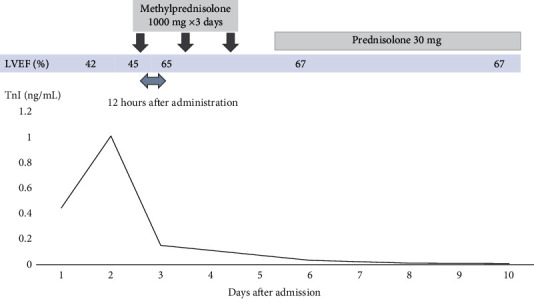
Changes in troponin I (TnI) level and left ventricular ejection fraction (LVEF) before and after steroid therapy. Rapid improvements in TnI and LVEF were found after the steroid administration.

On the next day after admission, peripheral blood eosinophil count decreased from 321/*μ*L to 162/*μ*L, whereas serum troponin I level continued to rise from 0.47 ng/*μ*L to 1.007 ng/*μ*L. Her symptoms (chest pain, nausea, and inappetence) persisted, and LVEF remained 45%. Although she was diagnosed with lymphocytic myocarditis, we decided to initiate steroid therapy because of the presence of eosinophil degranulation in the biopsy specimens. Methylprednisolone was administered at a dose of 1,000 mg/day for 3 days. Echocardiography performed 12 hours after the first administration of methylprednisolone showed marked improvement of LVEF from 45% to 65% (Figure 3) and normalization of regional wall motion. The pulse rate decreased and symptoms improved. On the following day, serum troponin I level decreased from 1.007 ng/*μ*L to 0.152 ng/*μ*L (Figure 3). Because of a dramatic response to high-dose steroid therapy, methylprednisolone was changed to oral prednisolone at a dose of 30 mg/day and tapered carefully. The troponin I level normalized by the eighth day after the first administration. The patient was discharged 20 days after admission with a normal LVEF and BNP level of 6.4 pg/mL. Paired serum viral antibodies for adenovirus, influenza virus, coxsackievirus, and enterovirus were all negative. The dose of oral prednisolone was tapered over 6 months to 2.5 mg/day and finally discontinued. There was no further recurrence of LV dysfunction or elevation of serum troponin I level.

## 3. Discussion

This report presents a case of acute lymphocytic myocarditis successfully treated with steroid therapy, in which eosinophil degranulation was observed in the abundant infiltration of lymphocytes into the myocardium. This case indicates that eosinophil degranulation in lymphocytic myocarditis may be an important finding associated with a high therapeutic response to steroid administration. Treatment strategy for acute myocarditis mainly focuses on supportive care to prevent heart failure or other fatal complications using inotropic agents or mechanical circulatory support [2, 4]. As for other pharmacological therapies, immunosuppressive therapy is thought to be effective for eosinophilic and giant cell myocarditis, which often has a fulminant presentation with abrupt impairment of LV function and lethal arrhythmia [1]. On the other hand, the benefits of immunosuppressive therapy in lymphocytic myocarditis still remain controversial [3]. In previous randomized controlled studies [5], immunosuppressive therapy did not improve the prognosis of acute lymphocytic myocarditis; therefore, routine steroid therapy is not recommended for lymphocytic myocarditis by the European Society of Cardiology guideline [2]. In contrast, several reports showed the effectiveness of steroid therapy in lymphocytic myocarditis. Reiff and Missov recently reported a case of fulminant lymphocytic myocarditis successfully treated with immunosuppressive therapy [6]. Weitsman et al. also reported on the effectiveness of early immunosuppressive therapy in acute lymphocytic myocarditis with persistent heart failure [7]. In these previous reports, the presence of virus and its association with clinical outcomes were mainly discussed, whereas the presence of eosinophil degranulation was not described. Furthermore, the presence and clinical implications of eosinophil degranulation in lymphocytic myocarditis have not been previously reported.

Previous in vitro studies have indicated that eosinophilic granule proteins, such as collagenase, major basic protein, and eosinophilic cationic protein, induce further migration of eosinophils and lead to progression of inflammation and myocardial damage [8]. Mast cells were also reported to be involved in inflammatory and fibrous response in the heart and be activated by eosinophilic granule proteins [9]. Furthermore, deMello et al. reported two clinical cases of acute necrotizing eosinophilic myocarditis in which deposition of the major basic protein of the eosinophil granule was identified within necrotic myocardium in autopsy specimens [10]. Therefore, we considered that eosinophil degranulation in myocarditis is an important finding which may be associated with progression of inflammation and myocardial damage. Because of the continuous rise in serum level of troponin I, which is an important marker of myocardial damage, further deterioration of cardiac function was of concern in the present case. Steroid therapy resulted in rapid and appreciable improvements not only in symptoms and vital signs, but also in cardiac function. Therefore, this can be considered a case of acute lymphocytic myocarditis with a high therapeutic response to steroid therapy. There may be several possible explanations for our findings. Combined lymphocytic and eosinophilic immune-mediated process may have been associated with progression of myocardial injury, in which early steroid administration may have regulated the eosinophilic immune-mediated process. Although viral infections have been considered the most important triggers of lymphocytic myocarditis, they may also cause eosinophilic myocarditis. In fact, Murphy et al. reported a case of eosinophilic-lymphocytic myocarditis after smallpox vaccination [11]. Another possibility is that eosinophil degranulation in the present case was an early sign of eosinophilic myocarditis and that early steroid therapy may have prevented progression of inflammation and myocyte necrosis due to further migration and degranulation of eosinophils. Eosinophilic myocarditis generally progresses from acute necrotic stage, marked by infiltration and extracellular deposition of eosinophils, to the fibrotic stage [12]. Myocyte necrosis has been known to be associated with eosinophil degranulation [8]. Myocardial specimens of our patient showed eosinophil degranulation with very little myocyte necrosis, which may have indicated that she was in a very early stage of eosinophilic myocarditis before myocardial necrosis developed.

Early diagnosis of eosinophilic myocarditis is sometimes difficult when endomyocardial biopsy specimens show no appreciable eosinophil infiltration. The presence of degranulation may be an initial important finding associated with the eosinophilic inflammatory process. It is noteworthy that a case of eosinophilia complicated by eosinophilic endomyocardial disease with deposition of eosinophil granule proteins in cardiac tissues in the virtual absence of eosinophil infiltration was previously reported [13].

In conclusion, early endomyocardial biopsy provides clinicians with valuable information about treatment strategy in patients with myocarditis. A close observation of biopsy specimens for eosinophil degranulation may help identify patients with lymphocytic myocarditis who may benefit from immunosuppressive therapy. Further investigation is required to pursue this possibility.
